# Efficacy of Immunization against a Novel Synthetic 13-Amino Acid Betaglycan-Binding Peptide Sequence of Inhibin α Subunit on Promoting Fertility in Female Rats

**DOI:** 10.3390/ijms24086914

**Published:** 2023-04-07

**Authors:** Xingfa Han, Xue Xia, Weihao Chen, Fengyan Meng, Xiaohan Cao, Guixian Bu, Tian Gan, Xiaogang Du, Qiuxia Liang, Xianyin Zeng

**Affiliations:** Isotope Research Lab, College of Life Science, Sichuan Agricultural University, Ya’an 625014, China

**Keywords:** inhibin, vaccine, folliculogenesis, ovulation, fertility, rat

## Abstract

Inhibins suppress the FSH production in pituitary gonadotrope cells by robustly antagonizing activin signaling by competitively binding to activin type II receptors (ACTR II). The binding of inhibin A to ACTR II requires the presence of its co-receptor, namely, betaglycan. In humans, the critical binding site for betaglycan to inhibin A was identified on the inhibin α subunit. Through conservation analysis, we found that a core 13-amino-acid peptide sequence <VRTTSDGGYSFKY> within the betaglycan-binding epitope on human inhibin α subunit is highly conserved across species. Based on the tandem sequence of such a conserved 13-amino-acid betaglycan-binding epitope (INHα13AA-T), we developed a novel inhibin vaccine and tested its efficacy in promoting female fertility using the female rat as a model. Compared with placebo-immunized controls, INHα13AA-T immunization induced a marked (*p* < 0.05) antibody generation, enhanced (*p* < 0.05) ovarian follicle development, and increased ovulation rate and litter sizes. Mechanistically, INHα13AA-T immunization promoted (*p* < 0.05) pituitary *Fshb* transcription and increased (*p* < 0.05) serum FSH and 17β-estradiol concentrations. In summary, active immunization against INHα13AA-T potently increased FSH levels, ovarian follicle development, ovulation rate and litter sizes, thus causing super-fertility in females. Therefore, immunization against INHα13AA is a promising alternative to the conventional approach of multiple ovulation and super-fertility in mammals.

## 1. Introduction

Inhibins are heterodimeric members of the transforming growth factor β (TGFβ) family, comprising a common α subunit that is disulfide-linked to either the inhibin βA subunit (inhibin A) or inhibin βB subunit (inhibin B) [1]. Inhibin A and inhibin B are endocrine hormones that are produced primarily by ovarian granulosa cells in females and by testicular Sertoli cells in males [1]. In female mammals, inhibin A and inhibin B are secreted across estrous cycles in a discordant pattern, with the dominant ovarian follicle and corpus luteum producing inhibin A, whereas smaller follicles secrete inhibin B [2]. Both inhibin A and inhibin B act as negative feedback regulators to selectively suppress follicle-stimulating hormone (FSH) production by the pituitary gonadotrope cells. FSH regulates the cyclic recruitment of small antral follicles, stimulating their growth and maturation to the preovulatory stage, meanwhile promoting 17β-estradiol synthesis within granulosa cells, and therefore playing essential roles in female fertility [3]. The blockage of inhibin bioactivities to improve follicular development and ovulation rate by increasing FSH secretion is a promising means to improve the fertility of females [4,5,6]. 

Both in vitro and in vivo studies evidenced that both inhibin A and inhibin B do not generate intracellular signals, and they mechanistically impair FSH biosynthesis by blocking activin signaling [3,7]. Activins are formed by homodimers or heterodimers of two inhibin β-subunits and potently stimulate FSH synthesis in pituitary gonadotrope cells through complexes of type II and I serine/threonine kinase receptors [1]. Specifically, activins bind to the type II receptors and trans-phosphorylate the type I receptors, which, in turn, phosphorylate the intracellular signaling protein SMAD family member 3 (SMAD3) [1]. Activated pSMAD3 then associates with SMAD4, translocates into the nucleus, and together with forkhead box L2 (FOXL2), binds to the proximal promoter of the FSHβ subunit gene (*Fshb*) [3]. Transcription of *Fshb* is the rate-limiting step in dimeric FSH biosynthesis. Inhibins robustly antagonize activin signaling by competitively binding to activin type II receptors via the β subunits they share with activins but do not phosphorylate and recruit the signaling type I receptors [1]. 

Subsequent studies further discovered that the binding of inhibin A and inhibin B to activin type II receptors requires the presence of their respective co-receptors [3,7]. Specifically, inhibin A antagonism of activins is dependent upon interactions with TGFβ type III receptor (TGFBR3, also known as betaglycan) [3,8], whereas inhibin B acts preferentially through an alternate transmembrane co-receptor, which is termed TGFβ receptor type III-like (TGFBR3L), to antagonize activin signaling [7]. Betaglycan and TGFBR3L directly bind to inhibin A and B, respectively, and promote the formation of a stable high-affinity complex with activin type II receptors to competitively antagonize activin-mediated receptor activation and *Fshb* transcription [3,7]. Conversely, mice with gonadotrope-specific betaglycan or knocked-out TGFBR3L were super-fertile, exhibiting increased folliculogenesis, numbers of ovulated eggs per cycle and litter sizes relative to controls [3,7].

Recently, an epitope critical for inhibin A binding to betaglycan was detected spanning the outer convex surface of the inhibin α subunit in humans [9]. Further homology modeling indicated that key inhibin α subunit residues containing Val^108^, Thr^111^, Ser^112^, Phe^118^, Lys^119^ and Tyr^120^ formed a contiguous epitope in this region of the molecule, and simultaneous mutation of Thr^111^, Ser^112^ and Tyr^120^ to alanine within this epitope region significantly abrogated binding affinity of inhibin A for betaglycan, and thus, its capacity to suppressing activin-stimulated FSH synthesis [9]. The discovery of this binding epitope provides a novel potential immune target to regulate FSH synthesis and thereby fertility in females.

Through conservation analyses, we found that a 13-amino acid sequence <VRTTSDGGYSFKY> within the betaglycan-binding epitope on human inhibin α subunit is highly conserved across species (see Section 4). Therefore, it appears that this core 13-amino acid epitope on the inhibin α subunit is the essential binding site of inhibin A with betaglycan across species. Based on this short 13-amino acid epitope, we designed and developed a novel inhibin vaccine. Using a rat model, we confirmed its good efficacy in promoting FSH-dependent ovarian follicular development, ovulation rate and fertility in females.

## 2. Results

### 2.1. INHα13AA-T Immunization Triggered Good Immunological and Biological Responses in Rats

**Figure 1:**
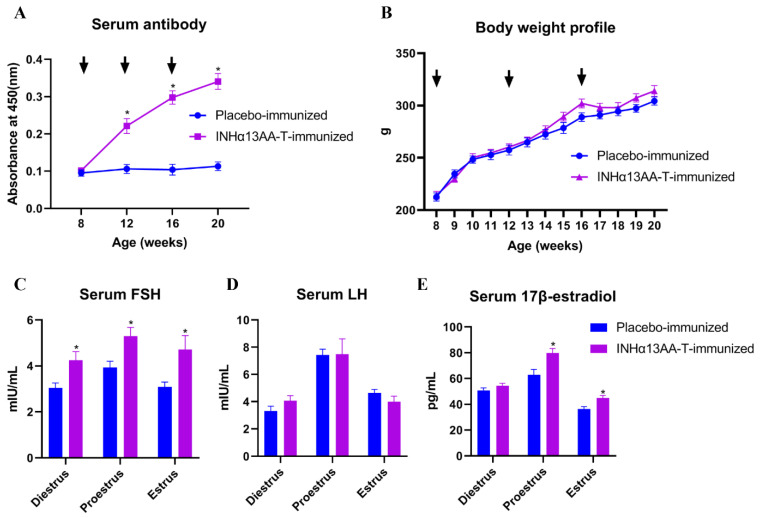
Effects of INHα13AA-T immunization on the reproductive physiology of female rats. (**A**) Serum anti-INH antibody titers (absorbance at 450 nm) in female rats immunized against INHα13AA-T-OVA. Microwell plates were coated with 5 µg INHα13AA-T-C in each well and incubated with sera collected from female rats immunized against INHα13AA-T-OVA or given a placebo emulsion. (**B**) Body weight profile of female rats following INHα13AA-T immunization. (**C**–**E**) Serum concentrations of FSH, LH and 17β-estradiol at decapitation. Arrows indicate the time point of vaccine injection. * *p* < 0.05 compared with placebo-immunized controls.

To evaluate the effect of INHα13AA-T immunization on immunological and biological response in female rats, the serum anti-inhibin antibody titers and body weight of female rats were monitored throughout the experimental period, and serum concentrations of FSH, LH and 17β-estradiol were determined at decapitation. Resultantly, INHα13AA-T immunization induced a good antibody response, especially after the booster immunizations (*p* < 0.05; Figure 1A), but exerted no significant effects on the body weight profile (*p* > 0.05; Figure 1B). In response to the sharp increase in serum inhibin-specific antibodies, the serum concentrations of FSH at diestrus, proestrus and estrus, as well as 17β-estradiol at proestrus and estrus, were increased (*p* < 0.05; Figure 1C,E) in INHα13AA-T-immunized females compared with placebo-immunized controls at decapitation. However, the serum LH concentrations were comparable between INHα13AA-T- and placebo-immunized rats at decapitation (*p* > 0.05; Figure 1D).

### 2.2. INHα13AA-T Immunization Prolonged Estrous Cycle Phases

**Figure 2:**
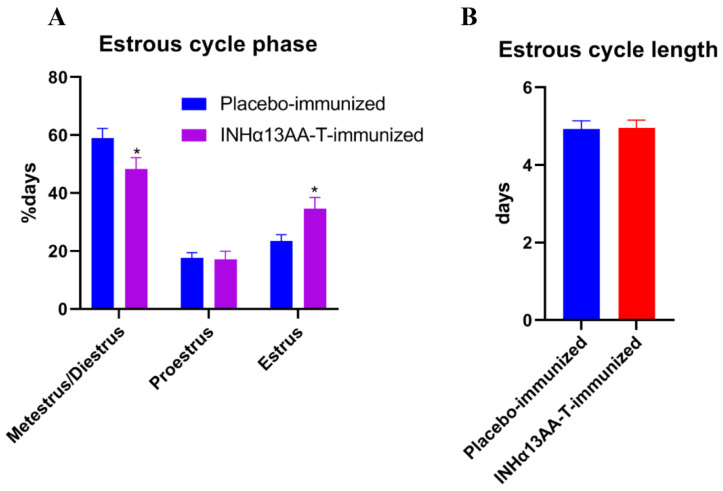
Effects of INHα13AA-T immunization on the estrous cycle of female rats. (**A**) Frequency of occurrence of cycle stages in female rats. The stage of the estrous cycle was determined by the predominant presence of nucleated epithelial cells (proestrus), cornified epithelial cells (estrus) or leukocytes (metestrus or diestrus). (**B**) Estrous cycle length of female rats. * *p* < 0.05 compared with placebo-immunized controls.

Based on vaginal cytology for 14 consecutive days starting 2 weeks after the second booster immunization, INHα13AA-T immunization shortened the duration of metestrus/diestrus phases (*p* < 0.05), but prolonged estrus phases (*p* < 0.05; Figure 2A). However, the estrous cycle length was not changed by INHα13AA-T immunization (*p* > 0.05; Figure 2B).

### 2.3. INHα13AA-T Immunization Increased the Thymus Weight and Index but Without Effects on Other Organs in Female Rats

**Figure 3:**
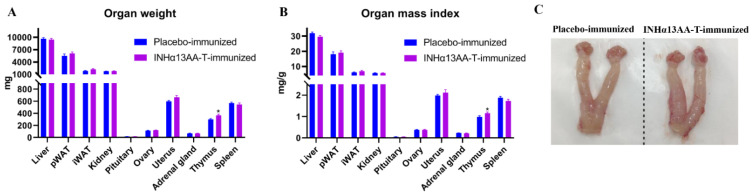
Effects of INHα13AA-T immunization on the weight and index of various organs in female rats. (**A**) The weight of various organs in placebo- and INHα13AA-T-immunized rats at decapitation. (**B**) The mass index of various organs in placebo- and INHα13AA-T-immunized rats at decapitation. (**C**) Representative photomicrographs of the ovary–oviduct–uterus complexes from placebo- and INHα13AA-T-immunized rats at decapitation. * *p* < 0.05 compared with placebo-immunized controls.

The weights of various organs and their relative weight (i.e., organ index) were measured and calculated at decapitation. Compared with placebo-immunized controls, INHα13AA-T immunization increased (*p* < 0.05) the thymus weight and index but exerted no effects on the weight or index of any other measured organs, including liver, pWAT, iWAT, kidney, pituitary, adrenal gland and spleen (Figure 3A,B). In particular, neither the weight nor the index of important reproductive organs, ovary and uterus were affected by INHα13AA-T immunization (*p* > 0.05; Figure 3A–C).

### 2.4. INHα13AA-T Immunization Promoted Ovarian Folliculogenesis and Natural Ovulation in Female Rats

**Figure 4:**
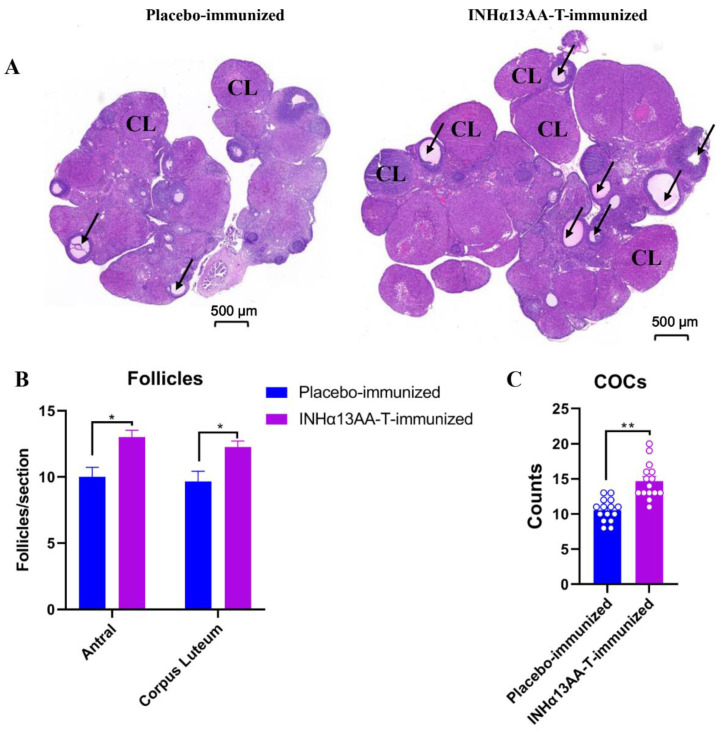
Effects of INHα13AA-T immunization on ovarian follicle development and ovulation of female rats. (**A**) Representative images of ovary sections from placebo- or INHα13AA-T-immunized rats. (**B**) The number of antral follicles (arrow) and corpora lutea (CL) in INHα13AA-T-immunized rats. (**C**) The number of COCs counted on the morning after mating. * *p* < 0.05 and ** *p* < 0.01 compared with placebo-immunized controls.

The efficacy of INHα13AA-T immunization in promoting folliculogenesis and ovulation in female rats was assessed at decapitation. Compared with placebo-immunized controls, there was a clear increase (*p* < 0.05) in the number of both antral follicles and corpora lutea (CL) in female rats following INHα13AA-T immunization (*p* < 0.05; Figure 4A,B). To directly assess the effects of INHα immunization on natural ovulation, we counted the cumulus–oocyte complexes (COCs) of female rats in the morning following mating. Consequently, compared with placebo-immunized controls, INHα13AA-T immunized females ovulated more eggs in natural cycles (*p* < 0.01; Figure 4C).

### 2.5. INHα13AA-T Immunization Promoted Pituitary Fshb and Ovarian Steroidogenesis- and Folliculogenesis-Associated Gene Expression

**Figure 5:**
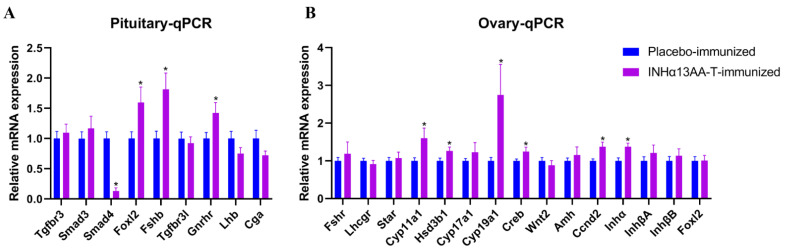
Effects of INHα13AA-T immunization on the expression of folliculogenesis-associated genes in the pituitary–ovary axis in female rats. (**A**) Relative expression of genes involved in folliculogenesis in the pituitary. (**B**) Relative expression of genes involved in folliculogenesis in ovaries. Abbreviations: *Tgfbr3*, TGFβ receptor 3; *Smad3*, SMAD family member 3; *Smad4*, SMAD family member 4; *Foxl2*, forkhead box L2; *Fshb*, follicle-stimulating hormone beta subunit; *Tgfbr3l*, TGFβ receptor type III-like; *Lhb*, luteinizing hormone beta subunit; *Cga*, glycoprotein hormones, alpha polypeptide; *Fshr*, follicle-stimulating hormone receptor; *Lhcgr*, luteinizing hormone receptor; *Star*, steroidogenic acute regulatory protein; *Cyp11a1*, cytochrome P450 family 11 subfamily A member 1; *Hsd3β1*, 3beta-hydroxysteroid dehydrogenase type 1; *Cyp17a1*, cytochrome P450 family 17 subfamily A member 1; *Cyp19a1*, cytochrome P450 family 19 subfamily A member 1 (aromatase); *Creb*, cAMP response element-binding protein; *Wnt2*, wingless-type MMTV integration site family, member 2; *Amh*, anti-Mullerian hormone; *Ccnd2*, cyclin D2; *Inha*, inhibin a; *InhβA*, inhibin beta subunit A; *InhβB*, inhibin beta subunit B. * *p* < 0.05 compared with placebo-immunized controls.

In accordance with increased circulating FSH concentrations in INHα13AA-T-immunized females, the mRNA expression of follicle-stimulating hormone beta subunit (*Fshb*) and *Fshb* upstreaming transcription regulators, including forkhead box L2 (*Foxl2*) and gonadotropin-releasing hormone receptor (*Gnrhr*), in the pituitary were markedly upregulated by INHα13AA-T immunization (*p* < 0.5, Figure 5A). Meanwhile, the mRNA expression of SMAD family member 4 (*Smad4*) was downregulated by INHα13AA-T immunization (*p* < 0.5; Figure 5A). Furthermore, mRNA expression of other measured upstream regulators of *Fshb* transcription, including SMAD family member 3 (*Smad3*) and transforming growth factor beta receptor 3 (*Tgfbr3*), in the pituitary were not affected by INHα13AA-T immunization (*p* > 0.5, Figure 5A). Moreover, mRNA expression of luteinizing hormone β subunit (*Lhb*), glycoprotein hormone alpha subunit (*Cgr*) and inhibin B co-receptor encoding gene (*Tgfbr3l*) in the pituitary were also not affected by INHα13AA-T immunization (*p* > 0.5, Figure 5A).

In ovaries, compared with placebo-immunized controls, the mRNA expression of ovarian steroidogenesis-associated genes, including cytochrome P450 family 11 subfamily A member 1 (*Cyp11a1*); 3beta-hydroxysteroid dehydrogenase type 1 (*HSD3β1*) and aromatase (*Cyp19a1*); and follicle development-associated genes, including cAMP response element-binding protein (*Creb*), cyclin D2 (*Ccnd2*) and inhibin alpha (*Inhα*), were all upregulated by INHα13AA-T immunization. Except for the above, the mRNA expression levels of other detected genes involved in steroidogenesis, including steroidogenic acute regulatory protein (*Star*); cytochrome P450 family 17 subfamily A member 1 (*Cyp17a1*); and genes involved in follicle development, including wingless-type MMTV integration site family, member 2 (*Wnt2*), anti-Mullerian hormone (*Amh*), inhibin βA(*InhβA*), inhibin βB (*InhβB*) and forkhead box L2 (*Foxl2*), were comparable (*p* > 0.05) between INHα13AA-T- and placebo-immunized females.

### 2.6. INHα13AA-T Immunization Improved the Fertility of Female Rats

**Figure 6:**
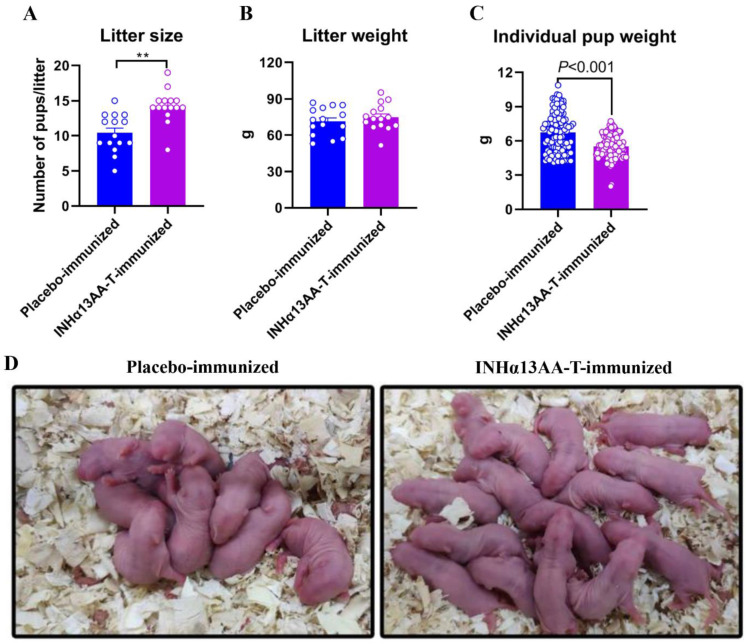
Efficacy of the effects of INHα13AA-T immunization on promoting fertility in female rats. (**A**) Litter size of pups born from placebo- or INHα13AA-T-immunized female rats. (**B**) Litter weight of pups born from placebo- or INHα13AA-T-immunized female rats. (**C**) Individual body weight of pups born from placebo- or INHα13AA-T-immunized female rats. (**D**) Representative photomicrographs of the litter sizes from placebo- or INHα13AA-T-immunized female rats. ** *p* < 0.01 compared with placebo-immunized controls.

To assess the efficacy of INHα13AA-T immunization on improving fertility in females, INHα13AA-T-immunized female rats and placebo-immunized controls were caged with males of proven fertility for 2 weeks, starting 4 weeks after the second booster immunization. Following these two weeks, all INHα13AA-T- and placebo-immunized rats were pregnant. Based on the breeding trial, INHα13AA-T-immunized female rats reproduced on average 3.8 more pups per litter than the placebo-immunized controls (*p* < 0.01; Figure 6A,D). However, the litter weight of pups was comparable between INHα13AA-T- and placebo-immunized rats (74.78 ± 2.82 versus 71.36 ± 2.96; *p* > 0.05; Figure 6B), with a significant decrease in the individual body weight of newborn pups of INHα13AA-T-immunized females than that of the placebo-immunized controls (6.72 ± 0.08 versus 5.76 ± 0.16; *p* < 0.05; Figure 6C).

## 3. Discussion

Global demand for meat products has increased dramatically in recent decades along with population growth. To cope with this big challenge, techniques for effectively increasing the ovulation rate and thereby fertility of farm animal species are urgently needed, especially for mono-ovulatory farm animals, such as sheep, goats and cattle. However, aside from defective exogenous gonadotropin protocols [10], no other fertility-improving approaches for farm animals have been practically applied so far. Based on the conserved betaglycan-binding peptide epitope on inhibin α subunit across species, we developed a novel inhibin vaccine. Using the female rat as a model, we demonstrated its good potency to increase circulating FSH levels and, consequently, ovarian follicle development, as well as the ovulation rate. Based on the breeding trial, INHα13AA-T immunized female rats produced on average 3.8 more pups per litter than their placebo-immunized controls. Therefore, active immunization against INHα13AA-based vaccines might be a very promising alternative to the conventional approach of multiple ovulation and super-fertility in females.

The majority of inhibin vaccines developed so far used immunogenic conjugates that incorporate synthetic peptides, which mimic the N-terminal sequence of the inhibin α subunit, such as incorporating the first bovine 26/29 [11,12], ovine 25/30 [13,14] or porcine 26/30/32 [15,16,17]. N-terminal amino residues of the inhibin α subunit. However, their animal biological responses were varied or even discrepant, and each peptide-based vaccine may be only effective within limited animal species [12,15,18,19]. Moreover, their action mechanisms also remained obscure [18]. Distinctly, our newly developed inhibin vaccine is based on the betaglycan-binding epitope on the inhibin α subunit. Given the indispensable role of betaglycan in facilitating the binding of inhibin A to activin type II receptors [3,9], antibodies generated from vaccines targeting betaglycan-binding epitope on inhibin α subunit would thus block the access of inhibin A to activin type II receptor and abrogate its functional antagonism of activin-mediated receptor activation and *Fshb* transcription. Indeed, the immunization of female rats against INHα13AA-T markedly enhanced pituitary *Fshb* transcription and increased serum FSH concentrations. To the best of our knowledge, this was the first study to demonstrate that the betaglycan-binding epitope on the inhibin α subunit could be used as a new hapten for developing novel inhibin antigens and vaccines. Given the betaglycan-binding epitope sequence on the inhibin α subunit is highly conserved across mammalian species, inhibin vaccines based on such peptide epitope sequence may therefore be effective for all mammalian species. However, further investigations and validations in this regard are still required.

Generally, there is a consensus that neutralizing endogenous inhibin would diminish its negative feedback regulation in the pituitary gland and, consequently, increase FSH production, thereby promoting folliculogenesis and fertility in females. Our results strongly supported this concept. That is, following INHα13AA-T immunization, we not only directly observed increased *Fshb* transcription in the pituitary and elevated FSH levels in serum at each estrus phase but also noticed significantly increased expressions of FSH-targeting genes, e.g., *Cyp19a1*, *Ccnd2* and *Inhα*, in ovaries. However, previous studies provided conflicting data. A large number of studies in both rodents and farm animals clearly indicated that active or passive immunization of animals against inhibin or inhibin α subunit increased pituitary FSH synthesis and secretion [11,12,14,15,19,20,21,22], but a few studies failed to detect increased plasma FSH concentrations in ruminants with increased ovarian follicular development and ovulation rate following inhibin immunization [13,17,18]. The reasons that caused this discrepancy between different studies remain obscure. Very interestingly, recent genetic manipulation studies in mice documented that the conditional knockout of either betaglycan [3] or TGFBR3L (inhibin B co-receptor) [7] in pituitary gonadotropes both augmented ovarian antral follicle development and litter size in the absence of a discernible alteration in pituitary FSH synthesis and secretion; meanwhile, complete blockage of both mature inhibin A and inhibin B generation by introducing a single inactivating point mutation in the inhibin α subunit (Arg233Ala) markedly increased serum FSH levels by two- to threefold and enhanced FSH-dependent follicle development and the natural ovulation rate [23]. These studies (1) provided compelling evidence that a pituitary-derived mechanism is responsible for the enhanced follicle development and ovulation rate in females when inactivating inhibin(s) because genetic manipulation was pituitary-gonadotrope-specific; (2) suggested that the absence of a discernible alteration in pituitary FSH synthesis/secretion when inactivating inhibin A might result from the compensatory augmentation of inhibin B actions and vice versa because the double-knockout of both inhibin A and inhibin B could markedly increased serum FSH levels while either knockout cannot [23]; and (3) implicated that the synthesis/secretion of pituitary FSH in response to inhibin inactivation is species-dependent, as neutralizing inhibin A in rats in our studies or other ruminant species in other studies [11,12,14,15,19,20,21,22] could increase pituitary FSH synthesis and secretion, but in mice, knocked-out inhibin A cannot [3]. Why the absence of discernible alteration of pituitary FSH synthesis/secretion after inhibin immunization or inhibin A knockout still could augment ovarian antral follicle development and fertility in females is unknown. Possibly, as suggested before [6], a small increase in circulating FSH concentrations could be sufficient to increase the ovulation rate. Taken together, a pituitary-derived and FSH-dependent mechanism is still the most likely one to cause multiple ovulation and super-fertility in females following inhibin immunization.

To elucidate how inhibin immunization increased FSH synthesis and secretion, we checked the transcriptional changes of all key genes within activin signaling in the pituitary. As a result, mRNA expression of forkhead box L2 (*Foxl2*) was increased, while SMAD family member 4 (*Smad4*) was decreased following INHα13AA-T immunization. Apart from this, no additional genes determined within activin signaling were transcriptionally affected. Forkhead box L2 is a well-known transcription factor that is essential for activin-stimulated *Fshb* transcription [24]. Its overexpression can potentiate activin induction of *Fshb* transcription [25], which was parallel to the enhanced transcription of *Fshb* in the pituitary. SMAD4 forms a complex with pSMAD3 and FOXL2 to finally activate the transcription of *Fshb* [3]. Its decreased expression created a paradox with the enhanced *Fshb* transcription. However, the mRNA expression level does not always correspond to the protein level, and its lower mRNA expression might represent a mechanism of negative feedback regulation, resulting from either its high protein levels or high pituitary FSH concentrations. Furthermore, we noticed that INHα13AA-T immunization significantly increased *Gnrhr* transcription in the pituitary as well. Consistently, mice with conditional knocked-out betaglycan from gonadotrope cells also exhibited enhanced mRNA expression of *Gnrhr* in the pituitary [3]. Increased *Gnrhr* expression in the pituitary may improve the FSH transcriptional response to GnRH. Therefore, the increase in FSH synthesis and secretion in INHα13AA-T-immunized females may involve synergistic interactions between activins and GnRH. Interestingly, the expression of both *Foxl2* and its target gene *Gnrhr* in gonadotropes was evidenced to be positively regulated by ovarian hormones [26]. Therefore, their enhanced expression in gonadotropes following INHα13AA-T immunization was at least partly attributed to increased ovarian hormone production, highlighting that increased pituitary FSH production in INH-immunized animals was also partially resulting from increased ovarian hormone production.

In ovaries, INHα13AA-T immunization substantially enhanced the folliculogenesis, as INHα13AA-immunized female rats contained more antral follicles and CL, ovulated more eggs in natural cycles, and had larger litter sizes than the controls. It was well established that FSH acts through FSH receptors on granulosa cells to induce ovarian steroidogenesis and follicle development, which are the two key regulators of folliculogenesis in females [27]. Meanwhile, both ovarian steroidogenesis and follicle development depend on the coordinated actions of FSH and LH [27]. In parallel to enhanced folliculogenesis, we found that the expressions of key genes associated with both follicle development (*Creb*, *Ccnd2* and *Inhα*) and estrogen biosynthesis (*Cyp11a1*, *HSD3b1* and *Cyp19a1*) in ovaries were all significantly upregulated in rats following INHα13AA-T immunization. Of these, except for *HSD3b1* in granulosa cells being directly regulated by FSH [28], the other genes were all directly FSH downstream genes [29,30]. In contrast, the LH target ovarian steroidogenic genes (*Star* [31] and *Cyp17a1* [32]), as well as pituitary *Lhb* expression and serum LH concentrations, were all unchanged, as was reported elsewhere [11,12,15,19,20,21,22]. Thus, the increased expressions of these key folliculogenesis-associated genes in ovaries were all driven by the increased secretion of pituitary FSH and independent of LH signaling, reinforcing the idea that INHα13AA-T immunization increased the folliculogenesis and ovulation rate almost exclusively through an FSH-dependent mechanism.

Except for enhanced folliculogenesis, we also noticed that INHα13AA-T immunization prolonged the estrus phase and shortened the metestrus/diestrus phases of female rats without an adverse effect on the estrous cycle length. It is clear that the recurrent estrous cycle is initiated and driven by the pulsatile secretion of GnRH from the hypothalamus [33]. INHα13AA-T immunization seemed to exert no effects on hypothalamic GnRH secretion rhythm and patterns, as evidenced by no change in pituitary LH. No change in the GnRH secretion rhythm and patterns following inhibin immunization or inactivation was also suggested elsewhere [3,6]. This may largely explain why INHα13AA-T immunization exerted no effects on the rat estrous cycle length. Meanwhile, the prolonged estrus phase in female rats following INHα13AA-T immunization might have been caused by the increased FSH secretion. In support, our previous studies in mice indicated that blocking FSH bioactivity by FSH immunization significantly shortened their estrous phase without affecting the estrous cycle length [34]. A longer estrus phase in females could facilitate and extend the period of their receptivity and readiness to mate the males [35], thus possibly leading to increased rates of conception.

For practical purposes, it is crucial to know whether inhibin immunization causes side effects in females. However, few studies performed previously have conducted such evaluations. In the present study, we systemically checked the effects of INHα13AA-T immunization on various organ weights and organ indexes of female rats. Resultantly, just from the organ weight/index standpoint, NHα13AA-T immunization appeared to exert no side effects on various determined organs, as neither their weight nor their index was changed by NHα13AA-T immunization. Intriguing, both the thymus weight and index in female rats were significantly increased by INHα13AA-T immunization. As a central immune organ, the thymus plays a crucial role in immunity and animal health by producing immune cells [36]. Whether the enlarged thymus can produce more immune cells and improve the immune function of INHα13AA-T-immunized females is unknown and deserves further studies. Moreover, the mechanism by which INHα13AA-T immunization increased the thymus weight and index also remains elusive and warrants further investigations.

In conclusion, the present study was the first to show that, using the female rat as a model, active immunization against the tandem form of conserved betaglycan-binding epitope <VRTTSDGGYSFKY> on inhibin α subunit led to increased pituitary FSH synthesis and secretion, and, in turn, increased folliculogenesis and the ovulation rate without pathological effects on the length of the estrus cycle, as well as the weight and index of various organs. Our findings demonstrate that active immunization against INHα13AA-T could be a useful method for improving the ovulation rate and fertility in females. Further evaluations on its direct efficacy in target farm animal species, such as sheep, goats and cattle, are required.

## 4. Materials and Methods

### 4.1. Homology Conservation Analysis of Betaglycan-Binding Epitope on Inhibin α Subunit

**Figure 7:**
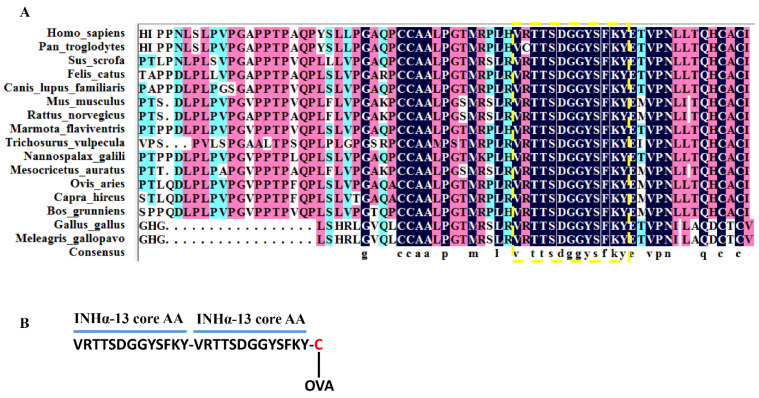
Conservation alignment of the betaglycan-binding epitope on inhibin α subunit across species. Makanji et al. [9] identified that in humans, the binding site of betaglycan for inhibin A is within a region containing a continuous 13-amino acid residue (marked by a yellow dotted box) on the inhibin α subunit. (**A**) Multiple sequence alignment using DNAMAN (version 9) indicates the 13-amino acid sequence within the betaglycan-binding epitope on inhibin α subunit is highly conserved across species. (**B**) Schematic illustrating the molecular design of the new inhibin antigen used in this study based on the conserved binding site of betaglycan for inhibin A.

Using inhibin A mutant proteins, an epitope for high-affinity betaglycan binding was identified that spanned the outer convex surface of the human inhibin α subunit [9]. Homology modeling and amino acid residue mutation demonstrated that the residues of Val^108^, Thr^111^, Ser^112^, Phe^118^, Lys^119^ and Tyr^120^ (especially Thr^111^, Ser^112^ and Tyr^120^) on inhibin α subunit are the key residues that are indispensable for inhibin A binding with betaglycan [9]. We downloaded the inhibin α subunit peptide sequence of the main vertebrate species from the NCBI website. Via conservation analysis using DNAMAN (version 9), we found that a 13-amino acid sequence <VRTTSDGGYSFKY> within the betaglycan-binding epitope region on the human inhibin α subunit is highly conserved across species, as marked by the dotted box in Figure 7A. Particularly, all the key amino acid residues, i.e., residues of Val^108^, Thr^111^, Ser^112^, Phe^118^, Lys^119^ and Tyr^120^, that were reported to interact with betaglycan in humans [9] were 100% conserved across all the analyzed species (Figure 7A), indicating that such a core 13-amino acid sequence (INHα-13AA) is the essential binding site of inhibin A with betaglycan across species. Therefore, vaccines or antibodies developed with this 13-amino acid peptide sequence could be used to block the binding of endogenous inhibin A with betaglycan, thus abrogating its functional antagonism of activin-mediated *Fshb* transcription and improving FSH-dependent fertility in female mammals. We chose the sequence with the highest consensus out of this 13-amino acid epitope sequence of inhibin α subunit across species, i.e., VRTTSDGGYSFKY (INHα13AA), as a basis hapten to develop a novel inhibin vaccine. Theoretically, antigens or vaccines developed with this 13-amino acid sequence should be effective at least in all the analyzed species herein.

### 4.2. Antigen Molecular Design, Synthesis and Inhibin Vaccine Formulation

To enhance INHα13AA immunogenicity and convert it to a complete antigen, INHα13AA was designed in tandem <VRTTSDGGYSFKY-VRTTSDGGYSFKY> (INHα13AA-T) with the addition of a cysteine at the C-terminal end (VRTTSDGGYSFKY-VRTTSDGGYSFKY-C, INHα13AA-T-C) to allow for its conjugation to the carrier protein (ovalbumin (OVA)) (Figure 7B). The synthesis of the novel INHα13AA-T-C peptide was conducted according to our previous descriptions [37]. Briefly, the peptide sequence of INHα13AA-T-C was first synthesized using standard Fmoc-based solid-phase peptide synthesis with 2-chlorotrityl chloride resin. Synthesis was performed on an ABI 430A peptide synthesizer. After synthesis, peptides were cleaved off the resin in a 95:2.5:2.5 trifluoroacetic acid (TFA)/triisopropylsilane (TIPS)/H2O mixture for 3 h and washed with dichloromethane. Crude peptides were purified via reverse-phase high-performance liquid chromatography (HPLC) using a Prep 2025 Column (20 mm × 250 mm, Bischoff, Leonberg, Germany) filled with Polygosil C18 material. The purity of the peptide was equal to 91.36% according to the peak area (Figure S1A). Pure fractions were collected and identified using ESI-MS (Figure S1B).

Subsequently, the purified peptide was conjugated to OVA via the –SH of cysteine at the C-terminal end of the peptide using maleimidobenzoyl-N-hydroxysulfosuccinimide ester (MBS), as described before [38]. Briefly, OVA (30 mg, Sigma) was dissolved in 0.1 M phosphate buffer (pH = 7.0) at a concentration of 15 mg.mL^-1^, the same volume of ethyleneglycol was added, followed by 70 μL of 0.125 M MBS solution in DMF. After stirring for 1 h, the solution was dialyzed (MW cut-off 10,000) three times for 30 min against a 0.1 M phosphate buffer (pH = 5.0). Lyophilized INHα13AA-T-C (30 mg) was added to the modified OVA and the mixture was shaken overnight at room temperature. The conjugate was dialyzed (MW cut-off 10,000) three times for 2 h against a 0.1 M phosphate buffer (pH = 5.0) and for 12 h against phosphate-buffered saline (PBS). The dialysate was lyophilized and stored at −80 °C pending emulsification. The loading was calculated based on comparative amino acid analysis of the conjugate and the separate peptide and carrier protein. According to the amino acid analyses, each milligram equivalent of INHα13AA-T-C-OVA was estimated to contain approximately 0.315 mg of INHα13AA-T-C that was covalently linked to 1.0 mg of OVA.

The vaccine emulsion was formulated as in our previous description [37]. In particular, INHα13AA-T-C-OVA was dissolved in 0.85% NaCl as in the water phase. Four parts of the water phase (45% (*v*/*v*)) were mixed with five parts (55% (*v*/*v*)) of Specol. The water phase was added to the oil phase (Specol) under continuous stirring using an Ultraturrax homogenizer (IKA, Staufen, Germany) running at 8000 rpm. Mixing was carried out until the emulsion displayed a homogeneous appearance.

### 4.3. Animals and Experimental Design

Sprague Dawley rats (Sichuan University Animal Center, Chengdu, China) were used for all experiments. Rats were housed in 12 h light/dark cycles at 25 ± 0.5 °C and 50–60% humidity with ad libitum access to standard chow and water.

### 4.4. INHα13AA-T Immunization Improved the Fertility of Female Rats

#### 4.4.1. Experiment 1: Effects of INHα13AA-T Immunization on the Reproductive Physiology and Natural Ovulation of Female Rats

Sixty female rats at 8 weeks of age with similar body weights were randomly allocated into two groups (*n* = 30). Thirty rats were actively immunized with INHα13AA-T emulsion (INHα13AA-T-immunized) at 8 wk of age with two booster injections at intervals of 4 weeks (same route and dose). For each immunization, each rat was given an intramuscular (IM) injection into a hind leg muscle with 0.5 mL vaccine emulsion containing 300 μg INHα13AA-T peptide equivalent of the conjugate. The remaining 30 rats (control) received placebo injections containing all components except INHα13AA-T-C-OVA. The body weights of the female rats were measured weekly, and blood for serum anti-inhibin antibody and reproductive hormone concentrations was sampled every 4 weeks from the tail tips until rats were deeply anesthetized with isoflurane (Fluriso; VetOne) and euthanized (decapitation) at 20 wk of age. The estrous cyclicity of all female rats was assessed for 14 consecutive days, starting 2 weeks after the second booster vaccination. Stages of the estrous cycle (proestrus, estrus, metestrus and diestrus) were determined using vaginal cytology, as described in [35].

At 20 wk of age, namely, 4 wk after the second booster vaccination, rats in each group were randomly allocated into two subgroups. Rats from one subgroup were euthanized to conduct physiological research, whereas rats from another subgroup were used to evaluate the efficacy of INHα13AA-T immunization on promoting natural ovulation. Rats used for physiological research were anesthetized with ether and then decapitated at morning estrus. After decapitation, various organs, including the liver, inguinal white adipose tissue (iWAT), periovarian white adipose tissue (pWAT), kidney, adrenal glands, thymus, spleen, pituitary, ovaries and uteri (two horns plus two cervices) were collected and weighed, and their relative weights (i.e., organ indexes) were calculated using organ weight/body weight. For each rat, the pituitary and right ovary were immediately frozen in liquid nitrogen and then stored at −80 °C for PCR analysis of gene expressions, and the left ovary was fixed in 10% buffered formalin for histological evaluation.

The efficacy of INHα13AA-T immunization in promoting natural ovulation of female rats was evaluated, as described previously [3]. In particular, INHα13AA-T- and placebo-immunized female rats were paired with male SD rats of proven fertility and inspected daily at 7 AM until a vaginal plug was visualized. Then, female rats were euthanized, and cumulus–oocyte complexes (COCs) were harvested in PBS from ampullae of the oviducts on both sides. Cumulus cells were dissociated from the oocytes by incubating with 0.5 mg/mL hyaluronidase (Sigma) for 10 min at 37 °C. Total oocyte numbers from each female rat were counted under an inverted microscope.

#### 4.4.2. Experiment 2: Effects of INHα13AA-T Immunization on Fertility of Female Rats

Thirty female rats at 8 wk of age with similar body weight were equally allocated to two groups (*n* = 15/group): INHα13AA-T-immunized and placebo-immunized. Rats were given INHα13AA-T or a placebo emulsion using the same vaccination program as described above. Four weeks after the second booster vaccination, INHα13AA-T- and placebo-immunized female rats were subjected to a mating study to assess their fertility. In brief, one INHα13AA-T-immunized rat and one placebo-immunized control were caged together with one male rat of proven fertility for 2 weeks, and then the male rat was removed. Litter size, litter weight and individual newborn pup’s body weight were recorded.

### 4.5. Antibody Titer Assays

Circulating anti-inhibin antibody titers in serum were determined using an enzyme-linked immunoabsorbent assay (ELISA). The 96-well plates (Thermo Electron Corporation, Waltham, MA, USA) were coated with INHα13AA-T-C peptide (5 µg each well) overnight at 4 °C. Plates were washed three times with PBS containing tri-(hydroxymethyl)-aminomethane (PBS-T) and the remaining binding sites were blocked by coating with 300 µL of 5% (*w*/*v*) skim milk powder (Molico Skim Milk, Nestlé, Araçatuba, SP, Brazil) in PBS-T buffer at 37 °C for 30 min. After three washes with PBS-T, 50 μL serum was added and incubated at 37 °C for 1 h. In addition, two negative controls (buffer without rat serum and pre-vaccination rat serum) were incubated on each plate. Plates were washed again with PBS-T, followed by the addition of 100 µL of 1:30,000 dilution horseradish peroxidase-conjugated goat anti-rat IgG (Sigma-Aldrich, St.Louis, MO, USA). After 1 h incubation at 37 °C, plates were washed three times in PBS-T and 3,3′,5,5′-tetra-methyl-benzidine (TMB; Sigma–Aldrich, St.Louis, MO, USA) was added. Plates were incubated for an additional 15 min before the reaction was stopped with sulfuric acid (2 M) and the absorbance read was at 450 nm with an ELISA plate reader (Tecan Sunrise, Tecan, Switzerland). Intra- and inter-assay CVs were calculated to be 8.5% and 12.7%, respectively.

### 4.6. Serum Hormone Assays

Circulating FSH and LH concentrations were determined using an enzyme-linked immunosorbent assay (ELISA), exactly as in our previous descriptions [39]. Circulating 17β-estradiol concentrations in serum were determined using commercial enzyme-linked immunosorbent assay kits (Cat#KGE014, R&D systems Inc., Minneapolis, USA) according to the manufacturer’s instructions. Assay sensitivities were 1.0 mIU/mL for both FSH and LH and 12.1 pg/mL for 17β-estradiol.

### 4.7. Ovary Histology and Follicle Counts

Ovaries from placebo- and INHα13AA-T-immunized rats were collected and fixed in 10% buffered formalin for 48 h, and then paraffin-embedded. Then, the entire ovary was serially sectioned at 5 μm and subjected to standard H&E staining. Quantification of the antral follicles and corpora lutea (CL) was done in every fifth section for each ovary. Follicles were classified according to our previous description [34]. In particular, the antral follicles were characterized by the formation of a fluid-filled cavity, and corpora lutea consisted of luteinized follicular mass. The average number of follicles per ovary was calculated by dividing the total number of follicles at a specific stage by the number of sections, as described in [40].

### 4.8. Relative Real-Time PCR (RT-PCR)

Total RNA was isolated from the pituitary and ovaries according to the manufacturer’s instructions (Invitrogen Co., Carlsbad, CA, USA). Quantitative and qualitative analyses of isolated RNA were assessed from the ratio of absorbance at 260 and 280 nm and agarose gel electrophoresis. A total of 1000 ng RNA was converted into first-strand cDNA using a PrimeScript^®^ RT reagent kit with a gDNA Eraser (TaKaRa Bio, Co., Ltd., Dalian, China). The RT-PCR was undertaken on a CFX96 Real-Time PCR detection system (BioRad, Hercules, California, USA). The PCR reaction contained 1 μL cDNA, 500 nmol/L each of forward and reverse primers, and 2×SYBR^®^ premix TaqTM (TaKaRa Bio Co., Ltd., Dalian, China). Primer sequences of target and reference genes are shown (Table S1). The PCR cycling conditions were as follows: initial denaturation at 95 °C (1 min), followed by 40 cycles of denaturation at 95 °C (5 s), annealing at 60 °C (25 s) and a final melting curve analysis (to monitor the PCR product purity). A reference housekeeping gene (*Gapdh*) was measured for each sample. The amplification efficiency of each RT-PCR primer, measured using a standard curve method, was within 90–110%. The fold change of mRNA in the treatment group relative to the control group was determined using 2^-ΔΔCt^.

### 4.9. Statistical Analyses

Statistical analysis was performed using GraphPad Prism 9.2 (La Jolla, CA, USA) software. Comparisons between two groups were carried out via unpaired two-tailed Student’s *t*-test. For the analysis of the effect of treatment in repeated measures (i.e., serum anti-FSH antibody and body weight), two-way ANOVA followed by Sidak’s multiple comparisons test was used. All values were expressed as the mean ± SEM and statistical significance was defined as *p* < 0.05.

## Data Availability

All data generated or analyzed during this study are included in this published article and its Supplementary Materials.

## Supplementary Materials

The following supporting information can be downloaded at: https://www.mdpi.com/article/10.3390/ijms24086914/s1.
