# Supplementary material for: Efficacy of Immunization against a Novel Synthetic 13-Amino Acid Betaglycan-Binding Peptide Sequence of Inhibin α Subunit on Promoting Fertility in Female Rats
Source: Int J Mol Sci. 2023 Apr 7;24(8):6914. doi: 10.3390/ijms24086914 (PMC10138769; doi:10.3390/ijms24086914)
Supplement: Supplementary file 1 [file ijms-24-06914-s001.zip › ijms-2266183-supplementary.pdf]

Table S1. RT-PCR Primer sequences for tissue genes.

Gene	Genbank accession no.	Primer sequence (5′-3′)	Amplification length (bp)
Tgfbr3	XM_039091792.1	F: CGGCTTTGGAAAAGAGAGTG<br>R: CAGGAGGAATGGTGTGGACT	215
Smad3	XM_039080898.1	F: TGGACGCAGGTTCTCCAAAC<br>R: CCGGCTCGCAGTAGGTAAC	90
Smad4	XM_032886206.1	F: GGCTGGTCGGAAAGGATT<br>R: GTGGGTAAGGATGGCTGT	270
Foxl2	XM_003750571.5	F: TCGCTAAGTTCCCGTTCTAC<br>R: GTAATTGCCCTTCTCGAACA	176
Fshβ	NM_001007597.2	F: CATCCTACTCTGGTGCTT<br>R: CACTCTTCCTTCTCTACTGA	84
Tgfbr3l	NM_001401205.1	F: CCTGACACCAGTGCCTTTGA<br>R: CTAGGGGACGGACGAGGTAT	156
Gnrhr	XM_032916489.1	F: TCTGCAATGCCAAAATCATC<br>R: GTAGGGAGTCCAGCAGATGAC	164
Lhβ	XM_032893648.1	F: CTTCTCCTCTTCTGATGC<br>R: TTTATTGGGAGGGATGGT	80
Cga	XM_032890134.1	F: CACTCTGGCATTTCCCATTA<br>R: GCCAGGTCCAAGAAGACAAT	108
Fshr	NM_199237.2	F: GAATGATGTCTTGGAAGTAATAG<br>R: CTTAATGCCTGTGTTGGA	163
Lhcgr	XM_039111807.1	F: TTACACATAACCACCATACC<br>R: TCCAGCGAGATTAGAGTC	134
Star	NM_031558.3	F: TTGGGCATACTCAACAACCA<br>R: ATGACACCGCTTTGCTCAG	389
Cyp11a1	XM_039080972.1	F: GGAGACATGGCCAAGATGGTA<br>R: TGATGCTGGCTTTGAGGAGTG	52
Hsd3b1	XM_032897642.1	F: TGAAAAATGGTGGCACACTGC<br>R: TATAGTTGTAAAATGGACGCAGC	414
Cyp17a1	NM_012753.3	F: ACTGAGGGTATCGTGGATGC<br>R: TCGAACTTCTCCCTGCACTT	161
Cyp19a1	NM_017085.3	F: TCCTCCTGATTCGGAATTGTG<br>R: GGCCCGATTCCCAGACA	70
Creb	XM_039084249.1	F: CCAAACTAGCAGTGGGCAGT<br>R: GAATGGTAGTACCCGGCTGA	140
Wnt2	XM_575397.8	F: TGGACAGCTGCGAAGTTATG<br>R: TGGACAGCTGCGAAGTTATG	263
Amh	NM_012902.2	F: GGAGAGACTGGGGAACAGC<br>R: CAAGAGCTGAGGCTCCCATA	69
Ccnd2	NM_022267.2	F: CAGAAGGACATCCAGCCGTAC<br>R: TCGGGACTCCAGCCAAGAA	136

Inhα	XM_032901415.1	F: GCCTTGGTCTCCTGCA<br>R: ACGCGTAGGGACCTCATGCTCC	300
InhβA	XM_008771712.3	F: TGGAGTGTGATGGCAAGGTC<br>R: AGCCACACTCCTCCACAATC	339
InhβB	NM_080771.2	F: TCTTCATCGACTTTCGGCTCAT<br>R: TGTCAGGCGCAGCCACACTCCT	304
Gapdh	XM_039097380.1	F: GGCAAGTTCAATGGCACAGT<br>R: TGGTGAAGACGCCAGTAGACTC	151

Abbreviations: Tgfbr3, TGFβ receptor 3; Smad3, SMAD family member 3; Smad4, SMAD family member 4; *Foxl2*, forkhead box L2; *Fshb*, follicle-stimulating hormone beta subunit; *Tgfbr3l*, TGFβ receptor type III-like; Lhb, luteinizing hormone beta subunit; *Cga*, glycoprotein hormones, alpha polypeptide; *Fshr*, follicle-stimulating hormone receptor; *Lhcgr*, luteinizing hormone receptor; *Star*, steroidogenic acute regulatory protein; *Cyp11a1*, cytochrome P450 family 11 subfamily A member 1; *Hsd3β1*, 3beta-hydroxysteroid dehydrogenase type 1; *Cyp17a1*, cytochrome P450 family 17 subfamily A member 1; *Cyp19a1*, cytochrome P450 family 19 subfamily A member 1(aromatase); *Creb*, cAMP response element-binding protein; *Wnt2*, wingless-type MMTV integration site family, member 2; *Amh*, anti-Mullerian hormone; *Ccnd2*, cyclin D2; *Inha*, inhibin a; *InhβA*, inhibin beta subunit A; *InhβB*, inhibin beta subunit B; *Gapdh*, glyceraldehyde-3-phosphate dehydrogenase.

A

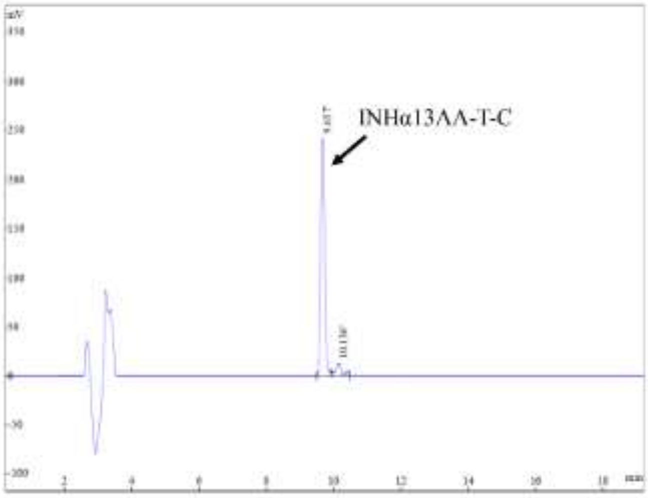

Rank	Time	Name	Conc.	Area
1	9.657		91.36	1865922
2	10.136		8.645	176572
Total			100	2042494

B

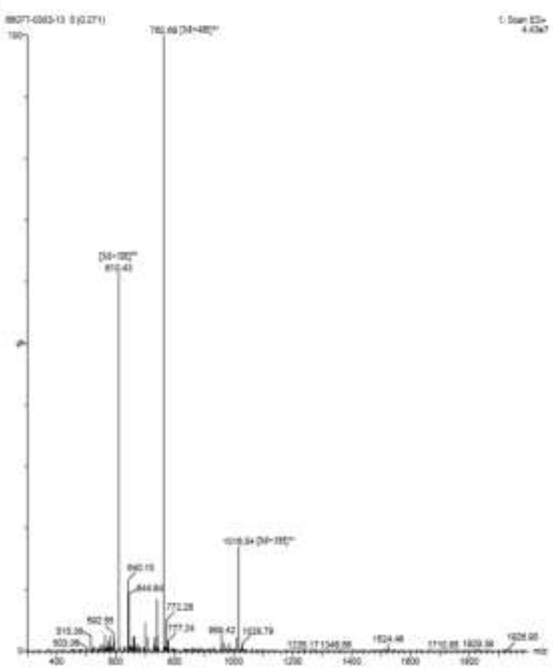

Figure S1. High-performance liquid chromatography (HPLC) purification of INHα13AA-T-C peptide. (**A**) According to the peak area, the purity of INHα13AA-T-C peptide was equal to 91.36%. (**B**) Identification of INHα13AA-T-C peptide using ESI-MS.
